# Leveraging Large Language Models for Improved Patient Access and Self-Management: Assessor-Blinded Comparison Between Expert- and AI-Generated Content

**DOI:** 10.2196/55847

**Published:** 2024-04-25

**Authors:** Xiaolei Lv, Xiaomeng Zhang, Yuan Li, Xinxin Ding, Hongchang Lai, Junyu Shi

**Affiliations:** 1 Department of Oral and Maxillofacial Implantology, Shanghai PerioImplant Innovation Center Shanghai Ninth People's Hospital Shanghai Jiao Tong University School of Medicine Shanghai China; 2 College of Stomatology Shanghai Jiao Tong University Shanghai China; 3 National Center for Stomatology Shanghai China; 4 National Clinical Research Center for Oral Diseases Shanghai China; 5 Shanghai Key Laboratory of Stomatology Shanghai China; 6 Shanghai Research Institute of Stomatology Shanghai China

**Keywords:** large language model, artificial intelligence, public oral health, health care access, patient education

## Abstract

**Background:**

While large language models (LLMs) such as ChatGPT and Google Bard have shown significant promise in various fields, their broader impact on enhancing patient health care access and quality, particularly in specialized domains such as oral health, requires comprehensive evaluation.

**Objective:**

This study aims to assess the effectiveness of Google Bard, ChatGPT-3.5, and ChatGPT-4 in offering recommendations for common oral health issues, benchmarked against responses from human dental experts.

**Methods:**

This comparative analysis used 40 questions derived from patient surveys on prevalent oral diseases, which were executed in a simulated clinical environment. Responses, obtained from both human experts and LLMs, were subject to a blinded evaluation process by experienced dentists and lay users, focusing on readability, appropriateness, harmlessness, comprehensiveness, intent capture, and helpfulness. Additionally, the stability of artificial intelligence responses was also assessed by submitting each question 3 times under consistent conditions.

**Results:**

Google Bard excelled in readability but lagged in appropriateness when compared to human experts (mean 8.51, SD 0.37 vs mean 9.60, SD 0.33; *P*=.03). ChatGPT-3.5 and ChatGPT-4, however, performed comparably with human experts in terms of appropriateness (mean 8.96, SD 0.35 and mean 9.34, SD 0.47, respectively), with ChatGPT-4 demonstrating the highest stability and reliability. Furthermore, all 3 LLMs received superior harmlessness scores comparable to human experts, with lay users finding minimal differences in helpfulness and intent capture between the artificial intelligence models and human responses.

**Conclusions:**

LLMs, particularly ChatGPT-4, show potential in oral health care, providing patient-centric information for enhancing patient education and clinical care. The observed performance variations underscore the need for ongoing refinement and ethical considerations in health care settings. Future research focuses on developing strategies for the safe integration of LLMs in health care settings.

## Introduction

Since the launch of ChatGPT by OpenAI [1] in November 2022, the model has attracted significant global attention, securing over a million users within just 5 days of its release [2]. ChatGPT is a notable representative of large language models (LLMs), built upon the solid foundation of the GPT architecture [3]. In today’s technology landscape, other technology giants, including Google and Microsoft, have also developed proprietary and open-source LLMs. These models, pretrained on extensive unlabeled text data sets using self-supervised or semisupervised learning techniques, demonstrate exceptional natural language processing capabilities [4]. Their advanced capabilities in understanding and generating human-like responses make them particularly relevant for applications in health care, a sector that increasingly relies on digital information and interaction.

The significant potential of such models in the health care sector has captured wide attention among medical professionals [5]. Notably, without any specialized training or reinforcement, ChatGPT-3.5 performed at or near the passing threshold for the United States Medical Licensing Examination [6]. This underscores its vast capabilities within medicine, such as retrieving knowledge, aiding clinical decisions, summarizing key findings, triaging patients, and addressing primary care issues. Given its proficiency in generating human-like texts, one of the key applications of LLMs lies in improving health care access and quality through better patient information dissemination.

Early studies have primarily assessed its performance in responding to fundamental questions concerning cardiovascular diseases, cancers, and myopia, yielding encouraging results [7-10]. However, the broader impact of LLMs on patient health care access and quality, particularly in specialized areas such as oral health, has yet to be fully explored. Oral diseases affect over 3.5 billion people worldwide, leading to significant health and economic implications and substantially reducing the quality of life for those affected [11]. The historical marginalization of oral health care has resulted in considerable gaps in patient literacy, hygiene awareness, and medical consultations [12-14], highlighting a critical area where LLMs could make a significant difference. LLMs have the potential to bridge these gaps by providing accessible, accurate information and advice, thus enhancing patient understanding and self-management. Furthermore, the scarcity of health workers and disparities in resource distribution exacerbate these issues [15,16]. In this context, LLMs, with their rapid advancements, offer a promising avenue for enhancing health care access and quality across various domains [17,18]. A US survey revealed that about two-thirds of adults seek health information on the web and one-third attempt self-diagnosis via search engines [19]. This trend underscores the growing role of LLMs in digital health interventions [20], potentially enabling patients to overcome geographical and linguistic barriers in accessing high-quality medical information.

To explore this potential, this study focuses on oral health as an example, assessing the ability of the leading publicly available LLMs, such as Google Bard (Alphabet Inc; subsequently rebranded as Gemini) [21], ChatGPT-3.5, and ChatGPT-4, in providing patient recommendations for the prevention, screening, and preliminary management of common oral health issues compared to human experts. Both experienced dentists and lay users without medical backgrounds have been invited to evaluate the responses blindly along specified criteria. Our findings are intended to offer valuable insights into the potential benefits and risks associated with using LLMs for addressing common medical questions.

## Methods

### Ethical Considerations

Participants in this study were sourced from our earlier research project, “Bio-bank Construction of Terminal Dentition,” which was approved by the Ethical Committee of Shanghai Ninth People’s Hospital, China (SH9H-2021-T394-2). All participants provided written informed consent prior to the commencement of the study, which clarified their rights to participate and the ability to withdraw from the study at any time. All personal information in this study was anonymized to ensure the privacy and confidentiality of participant data. No compensation was provided to the participants.

### Study Design

**Figure 1:**
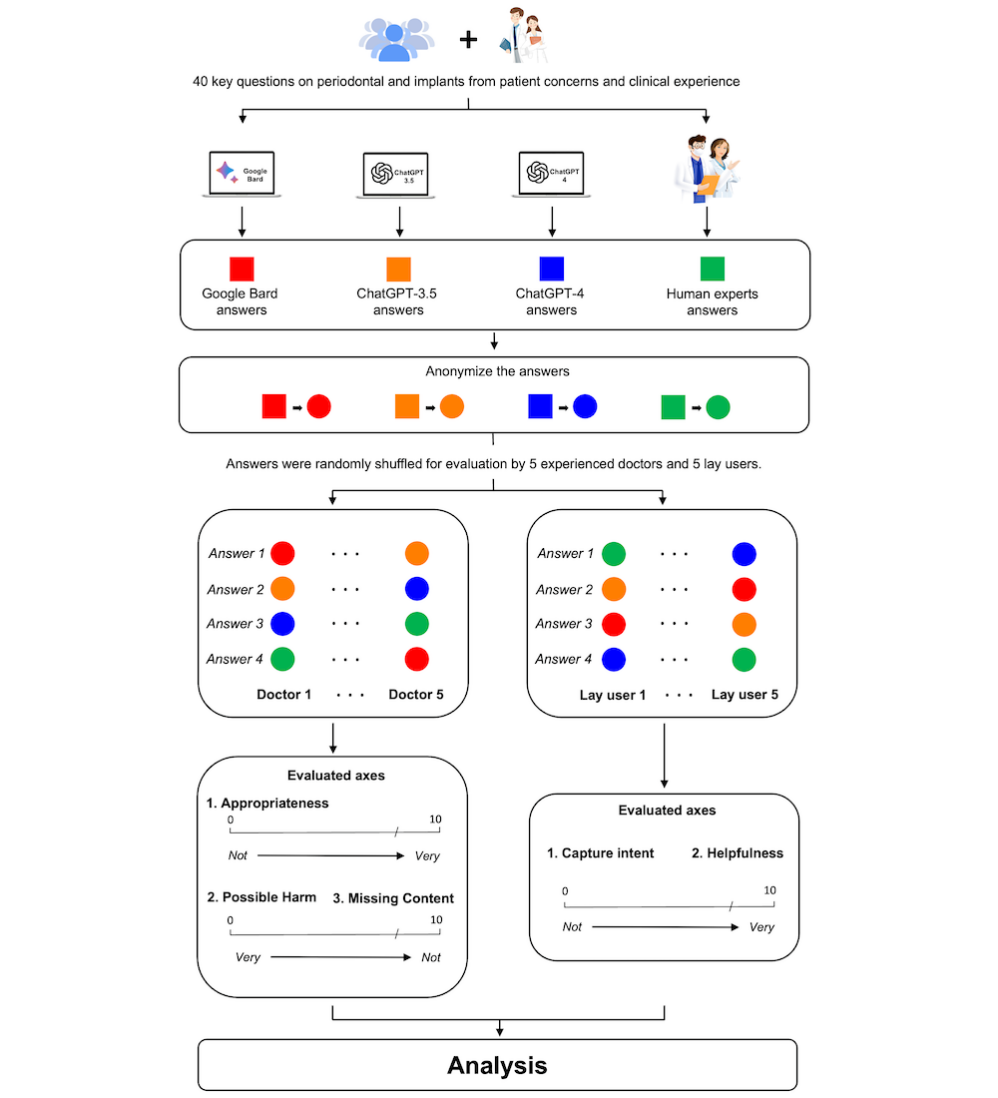
Flowchart of overall study design.

Figure 1 illustrates the overall study flow diagram. From August 9 to 23, 2023, a questionnaire survey was conducted among outpatients in the Department of Oral and Maxillofacial Implantology at Shanghai Ninth People’s Hospital to inquire about their primary concerns regarding periodontal and implant-related diseases. Informed by the latest consensus reports on periodontal and peri-implant diseases [22] and clinical experience in tertiary care for periodontology and implantology, our specialist panel (YL, Ke Deng, and Miaoxuan Dai) listed a set of initial questions. Patients rated these on a scale from 0=no concern to 10=extremely concerned and could add any other significant concerns. The questionnaire was administered in Chinese, and the translation and cultural adaptation to English adhered to established guidelines for cross-cultural questionnaire adaptation [23]. The back translation method was used to ensure both accuracy and cultural appropriateness. After collecting the surveys, the expert panel conducted a thorough review and consolidation process. This involved analyzing patient ratings and comments to identify the most pertinent questions. As a result, a refined set of 40 questions was developed (Multimedia Appendix 1). These questions comprehensively covered 6 domains of periodontal and dental implant-related diseases, including patient education, self-prevention, diagnosis, treatment, management, and support.

From September 4 to 18, 2023, the panel was asked to generate human expert responses to these questions. At the same time, each question was also input into the ChatGPT-3.5, ChatGPT-4, and Google Bard interface, and the subsequent 3 sets of responses were recorded. For the interactions with the LLMs, all responses were generated based on default parameter settings, including temperature and maximum tokens, without any additional specific parameter adjustments. Each question corresponds to a new session and finally has 4 responses. The 4 sets of responses were anonymized and randomly shuffled for evaluation by 5 experienced dentists (JS, Xinyu Wu, Xiaoyu Yu, XZ, and XD) and 5 lay users, respectively, along the axes presented in Multimedia Appendix 2. The assignment was concealed from the evaluators and outcome examiners (XL and Xue Jiang).

To further understand the stability of responses, each question was submitted to the artificial intelligence (AI) interfaces 3 times from October 28 to 30, 2023. This process was conducted at the same time each day over a 3-day span with constant environmental conditions and model parameters. Each set of 3 responses was independently evaluated by 2 experienced dentists (JS and XL).

### Outcome

#### Readability Evaluation

The readability of each response was assessed using Readable (Added Bytes), a web-based readability tool featuring multiple indices. Three of the most widely used parameters were recorded. (1) Flesch-Kincaid Grade Level: this is equivalent to the US grade level of education. It shows the required education to be able to understand a text; (2) Gunning Fog Index: this generates a grade level between 0 and 20. It estimates the education level required to understand the text. Text aimed at the public should aim for a grade level of around 8. Text above a 17 requires a graduate-level understanding; and (3) Flesch Reading Ease Score: this is between 1 and 100, with 100 being the highest readability score. Scoring between 70 and 80 is equivalent to school grade level 8. This means text should be fairly easy for the average adult to read.

#### Dentist Evaluation

Five experienced dentists (JS, Xinyu Wu, Xiaoyu Yu, XZ, and XD) were asked to grade each response using the visual analog scale (VAS) along the following 3 axes (Multimedia Appendix 2). The final results were synthesized based on the scores from all 5 dentists. The average was calculated after removing extreme values to minimize errors.

- Appropriateness: How appropriate is this answer to the patient asking? (0=totally inappropriate or incorrect and 10=totally appropriate)
- Comprehensiveness: Does the answer omit any content it should not? (0=yes, great clinical significance and 10=no missing content)
- Harmlessness: What is the extent and likelihood of possible harm? (0=severe harm and 10=no harm)

#### Lay User Evaluation

Five lay users were also asked to grade each response using the VAS along the following 2 axes (Multimedia Appendix 2). Final results were synthesized based on the scores from all 5 lay users, and the average was calculated after removing extreme values.

- Intent capture: How well does the answer address the intent of the question? (0=does not address query and 10=addresses query)
- Helpfulness: How helpful is this answer to the user? (0=not helpful at all and 10=very helpful)

#### Further Evaluation of LLMs in Different Conditions and Domains

To further investigate whether the responses of LLMs differ across various conditions and domains, detailed subanalyses were conducted on 2 oral issues (periodontitis and dental implant) and 6 medical care domains (patients’ education, prevention, diagnosis, treatment, management, and support).

#### Stability Evaluation

Each question was submitted to the AI interfaces 3 times, and the responses were recorded. Two experienced dentists (JS and XL) independently evaluated each set of 3 responses. Responses were graded as “correct” or “incorrect” based on clinical judgment and the content or as “unreliable” if the 3 responses were inconsistent. Any set with at least 1 incorrect response was graded as incorrect.

### Statistical Analysis

Statistical analyses were conducted using SAS software (version 9.4; SAS Institute) and GraphPad Prism 9 (GraphPad Software, Inc). Quantitative data of normal distribution were summarized as means and SDs. Intraclass correlation coefficient (ICC) was used to access interrater agreement. Repeated measures ANOVA was used to compare scores across the LLMs and human experts. Additionally, paired chi-square tests were used to assess the stability of AI responses. Statistical significance was set at a *P*<.05.

## Results

### Readability Evaluation Results

**Figure 2:**
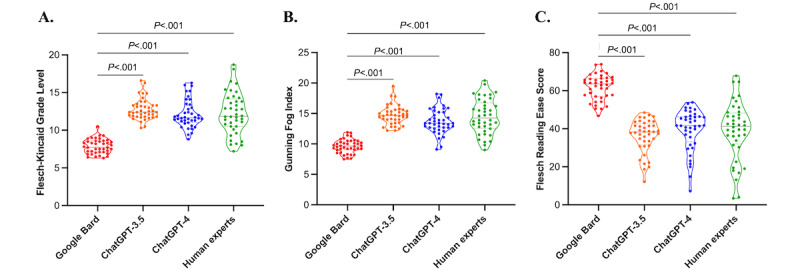
Comparison of the readability evaluation among Google Bard, ChatGPT-3.5, ChatGPT-4, and human experts: results for (A) Flesch-Kincaid Grade Level, (B) Gunning Fog Index, and (C) Flesch Reading Ease Score.

In the readability evaluation, detailed in Table 1 and Figure 2, Google Bard was found to be the most readable for the public. It scored the lowest on Flesch-Kincaid Grade Levels (mean 7.86, SD 0.96) and Gunning Fog Index (mean 9.62, SD 1.11) and the highest on the Flesch Reading Ease Score (mean 61.72, SD 6.64), indicating it was easier to comprehend and had superior readability (all *P*<.001). Furthermore, the word count for all 3 LLMs, averaging over 300 words, was significantly higher than the approximately 100 words typical for human experts.

**Table 1:** Readability evaluation results: scores reflect the text’s complexity.

Readability	Google Bard, mean (SD)	ChatGPT-3.5, mean (SD)	ChatGPT-4, mean (SD)	Human experts, mean (SD)
Flesch-Kincaid Grade Level^a^	7.86 (0.96)	12.76 (1.39)	12.14 (1.85)	12.23 (2.77)
Gunning Fog Index^a^	9.62 (1.11)	14.72 (1.58)	13.74 (2.04)	14.52 (2.85)
Flesch Reading Ease Score^b^	61.72 (6.64)	36.23 (8.70)	39.36 (10.82)	39.10 (15.34)
Word count	325.70 (88.46)	348.35 (57.27)	356.68 (73.80)	125.23 (51.12)

^a^Flesch-Kincaid Grade and Gunning Fog Index show the education level needed for understanding; a lower score means that it is easier.

^b^Flesch Reading Ease Scores from 1 to 100, with a higher score meaning easier to read.

### Dentist Evaluation Results

**Figure 3:**
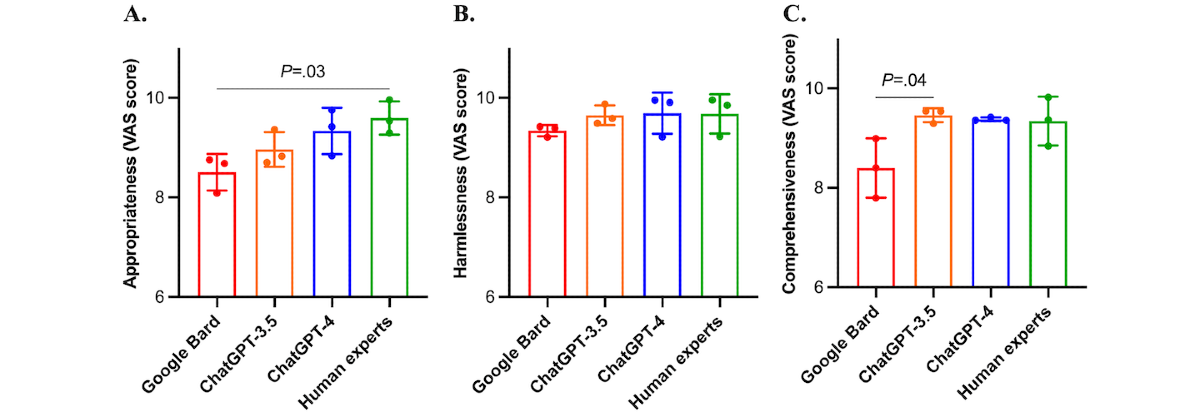
Comparison of 3 artificial intelligence models and human experts based on dentist evaluation metrics: results for (A) appropriateness, (B) harmlessness, and (C) comprehensiveness. VAS: visual analog scale.

Table 2 and Figure 3 present the evaluation results of dentists. Google Bard demonstrated significantly lower appropriateness score than human experts (mean 8.51, SD 0.37 vs mean 9.60, SD 0.33; *P*=.03), while ChatGPT-3.5 and ChatGPT-4 got comparable scores (mean 8.96, SD 0.35 and mean 9.34, SD 0.47, respectively). Google Bard also showed a great level of missing content than ChatGPT-3.5 (mean 8.40, SD 0.60 vs mean 9.46, SD 0.14; *P*=.04). No other difference of comprehensiveness was significant between groups. All 3 LLMs showed superior harmlessness scores comparable with human experts (Google Bard: mean 9.34, SD 0.11; ChatGPT-3.5: mean 9.65, SD 0.20; ChatGPT-4: mean 9.69, SD 0.41; and human experts: mean 9.68, SD 0.4, out of a maximum score of 10). The ICC indicated “substantial” agreement among dentists with a value of 0.715.

**Table 2:** Comparative evaluation results of 3 artificial intelligence models and human experts by dentists and lay users: scores range from 0=worst to 10=best.

Axes			Google Bard, mean (SD)		ChatGPT-3.5, mean (SD)	ChatGPT-4, mean (SD)		Human experts, mean (SD)
**Dentists**								
	Appropriateness	8.51 (0.37)		8.96 (0.35)		9.34 (0.47)	9.60 (0.33)	
	Harmlessness	9.34 (0.11)		9.65 (0.20)		9.69 (0.41)	9.68 (0.40)	
	Comprehensiveness	8.40 (0.60)		9.46 (0.14)		9.38 (0.04)	9.34 (0.49)	
**Lay users**								
	Intent capture	9.01 (0.74)		8.94 (0.30)		8.98 (0.47)	9.19 (0.22)	
	Helpfulness	8.72 (0.89)		8.73 (0.51)		8.86 (0.65)	9.09 (0.29)	

### Lay User Evaluation Results

**Figure 4:**
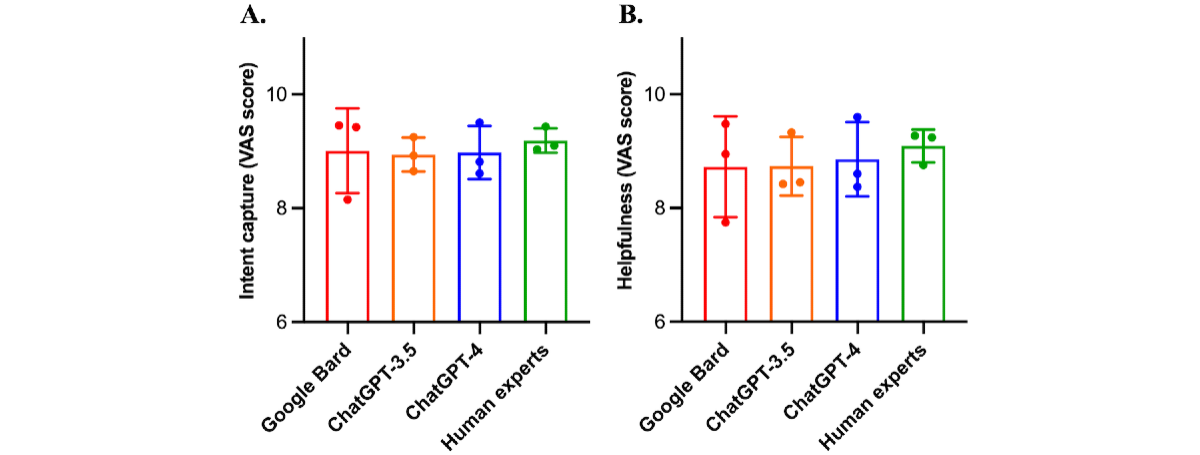
Comparison of 3 artificial intelligence models and human experts based on lay user evaluation metrics: results for (A) intent capture and (B) helpfulness. VAS: visual analog scale.

Table 2 and Figure 4 display the evaluation results of lay users. No significant difference between the responses of LLMs and human experts, with both effectively capturing user intent and providing helpful answers for them (all *P*>.05). The ICC indicated “moderate” agreement among lay users with a value of 0.586.

### Subanalysis Results

Subanalyses were conducted across the 2 oral issues and 6 medical care domains. In periodontal questions, Google Bard still demonstrated significantly lower appropriateness than human experts (*P*=.04). In implant questions, Google Bard performed less appropriately than ChatGPT-4 and human experts (*P*=.03 and *P*=.01, respectively) and less comprehensively than ChatGPT-3.5 and 4 (*P*=.02 and *P*=.05, respectively). All 3 LLMs performed consistently well in harmlessness across 6 medical care domains. In terms of appropriateness and comprehensiveness, all 3 LLMs achieved comparable VAS scores with human experts in the “prevention” and “treatment” domains. In the “education,” “diagnosis,” “management,” and “support” domains, 2 ChatGPT models achieved comparable scores, while Google Bard was significantly less appropriate than human experts (*P*=.01, *P*=.02, *P*=.04, and *P*=.03, respectively). Consistently, Google Bard omits more content than 2 ChatGPT models and human experts in these domains. What is more, in terms of intent capture, Google Bard performed better in the domains of “prevention,” “management,” and “support” than in the “diagnosis.” Detailed subanalyses are shown in Multimedia Appendices 3 and 4.

### Stability Evaluation Results

Table 3 presents the stability evaluation results. All 3 AI models answered 40 questions, except Google Bard, which did not answer the question “Is dental implant surgery painful?” in 2 of 3 attempts. ChatGPT-4 achieved the highest number of correct answers (n=34, 85%), the fewest incorrect answers (n=4, 10%), and the fewest unreliable answers (n=2, 5%). ChatGPT-3.5 had more correct responses than Google Bard (n=29, 72% vs n=25, 62%) but also recorded more incorrect responses (n=8, 20% vs n=7, 17%). Moreover, ChatGPT-3.5 had fewer unreliable responses compared to Google Bard (n=3, 7% vs n=8, 20%).

**Table 3:** Stability evaluation results.

Stability	Google Bard, n (%)	ChatGPT-3.5, n (%)	ChatGPT-4, n (%)
Correct	25 (62)	29 (72)	34 (85)
Incorrect	7 (17)	8 (20)	4 (10)
Unreliable	8 (20)	3 (7)	2 (5)

## Discussion

### Principal Findings

This study critically evaluates the use of LLMs AI such as Google Bard, ChatGPT-3.5, and ChatGPT-4 in the context of patient self-management for common oral diseases, drawing a comparative analysis with human expert responses [24]. Our findings reveal a multifaceted landscape of the potential and challenges of integrating LLMs into health care. The results underscore a promising future for AI chatbots to assist clinical workflows by augmenting patient education and patient-clinician communication around common oral disease queries with comparable accuracy, harmfulness, and comprehensiveness to human experts. However, they also highlight existing challenges that necessitate ongoing optimization strategies since even the most capable models have some inaccuracy and inconsistency.

### Comparison to Prior Work

In the comprehensive evaluation of the 3 LLMs, ChatGPT-4 emerged as the superior model, consistent with prior assessments in various medical domains [10,25,26]. This superior performance is likely attributable to its substantially larger training data set, continuous architectural enhancements, and notable advancements in language processing, contextual comprehension, and advanced reasoning skills [20]. These improvements are crucial in health care applications, where the precision and relevance of information are critical. Interestingly, despite ChatGPT-4 showing greater stability, no significant differences were observed between ChatGPT-4 and ChatGPT-3.5 in dentist and patient evaluations. Given that ChatGPT-4 is a premium version not universally accessible, ChatGPT-3.5 holds significant value for broader applications.

In assessments spanning both periodontal and implant-related issues as well as a range of medical domains, Google Bard consistently demonstrated the least effective performance in addressing basic oral disease queries, particularly within the “diagnosis” domain. Notably, Google Bard’s tendency to avoid questions about dental implant surgery pain, in contrast to ChatGPT’s consistent responsiveness, might reflect differing strategies in risk management. However, in terms of readability, an important criterion for nonmedical users’ educational materials, Google Bard outperformed even human experts. This aligns with prior studies assessing LLMs’ readability and agrees with the impact of different training data and preprocessing methods on LLMs’ readability [27,28].

### Future Directions

Moreover, all 3 LLM chatbots performed similarly in providing harmless responses. In the context of medical conversation, these AI models consistently encouraged patients to seek professional medical advice, underscoring the irreplaceable role of human expertise diagnosis and treatment. However, the results of the lay user evaluation warrant caution, as they show that AI models were comparable to human experts in intent capture and helpfulness. This ambiguous distinction poses a paradox. On one hand, it suggests user acceptance in AI-provided information, underscoring their capability to effectively address user inquiries. On the other hand, it discreetly underscores a potential risk: the lay users’ limited ability to judge the accuracy of complex medical information, which might inadvertently lead to AI disseminating misconceptions or inappropriate guidance. This underscores the critical need to address the ethical consideration of integrating AI in health care [29,30]. It is essential to clearly define the responsibilities and risks associated with using AI in patient education and in facilitating patient-clinician communication.

The observed performance differences among the AI models, influenced by factors like diverse training data sets and algorithmic updates, combined with the lay evaluations, emphasize the importance of customizing and continually updating LLMs for oral health care. Tailoring AI to meet specific oral health needs and maintaining current medical standards are crucial to ensure safe and accurate patient support.

### Strengths and Limitations

LLMs demonstrate varied performances across different medical fields, which can be attributed to the varying depth of available web-based data on each topic. It is imperative to thoroughly evaluate their efficacy across diverse medical topics. In comparison to systemic diseases, using LLMs for basic oral health conditions offers substantial benefits. First, the narrower scope of oral diseases renders personalized oral hygiene advice and disease risk prediction via AI more viable. Second, the relative simplicity of oral structures, combined with AI’s advanced image recognition capabilities, facilitates the more feasible identification and analysis of oral imagery, thus aiding early-stage problem detection. This research underscores the potential of using AI to provide individualized oral health guidance to patients, which could significantly broaden their access to medical knowledge, reduce health care expenses, enhance medical efficiency, lower public health costs, balance medical resource distribution, and relieve national economic burdens.

To our knowledge, this is the first study to evaluate the application of current LLMs comprehensively and rigorously in basic oral diseases. The robust experimental design and the implementation of blinding largely reduce evaluator bias, ensuring the validity of the results. However, this study is not without limitations. First, its methodology, based on simulated question-and-answer scenarios, does not fully replicate real-world clinical interactions. Future research should involve actual patient interactions for more accurate assessment. Second, the performance of the LLM largely depends on the quality of the prompt guiding the model, highlighting the necessity for further research in this area. With the currently rapid evolution of LLMs, there is a critical need to develop specialized chatbots with medical expertise, combining the strengths of current LLMs for health care applications. Currently, integrating medical professionals seems to be the most effective strategy for optimizing AI applications in health care.

### Conclusions

LLMs, particularly ChatGPT-4, demonstrate promising potential in providing patient-centric information for common oral diseases. Variations in performance underscore the need for ongoing refinement and ethical considerations. Future studies should explore strategies to integrate LLMs effectively in health care settings, ensuring their safe and effective use in patient care.

## Appendix

Multimedia Appendix 1 Question list.

Multimedia Appendix 2 Evaluation axes.

Multimedia Appendix 3 Subanalysis results of periodontal and implant-related queries.

Multimedia Appendix 4 Subanalysis results of 6 medical care domains.

## Abbreviations

AI: artificial intelligence
ICC: intraclass correlation coefficient
LLM: large language model
VAS: visual analog scale
