# Supplementary material for: Leveraging Large Language Models for Improved Patient Access and Self-Management: Assessor-Blinded Comparison Between Expert- and AI-Generated Content
Source: J Med Internet Res. 2024 Apr 25;26:e55847. doi: 10.2196/55847 (PMC11082737; doi:10.2196/55847)
Supplement: Multimedia Appendix 1 [file jmir_v26i1e55847_app1.pdf]

Questions	Domains
**Perio-questions**	
1. I've been diagnosed with periodontitis. What should I do at home?	D1, D5
2. What is periodontitis? How can I tell if I have periodontal disease?	D1, D3
3. What can I do at home to prevent periodontitis?	D2
4. My family members have periodontal disease. Does that increase my risk of developing periodontitis?	D1
5. I have a smoking habit. Does that increase my risk of developing periodontitis?	D1
6. I have diabetes and cardiovascular disease. Does that increase my risk of developing periodontitis?	D1
7. How often should I get a professional dental cleaning?	D2
8. If I don't have symptoms of periodontitis, do I still need regular check-ups?	D2
9. Are gum redness and bleeding while brushing indicate that I have periodontitis?	D3
10. My teeth are gradually becoming loose. Does this indicate that I have periodontitis?	D3
11. The doctor mentioned that the periodontal pocket probing depth of one of my teeth is 10mm. What does this indicate?	D1
12. Is it necessary to take antibiotics for periodontitis?	D4
13. Can periodontitis heal on its own?	D4
14. What are the treatment methods for periodontitis?	D4
15. Is it safe to undergo teeth cleaning during pregnancy?	D1, D2
16. Are there any side effects of teeth cleaning? Can it damage my teeth, make my gaps wider, or cause my teeth to become loose?	D1
17. The doctor mentioned that I need subgingival scaling. What is subgingival scaling?	D1, D4
18. How much does it cost to get a dental cleaning in RMB?	D1
19. How long does a typical periodontal treatment cycle last?	D4
20. Is it necessary to have follow-up appointments after the treatment is completed?	D5

Dental implant-questions		
1.	What is a dental implant?	D1
2.	Is everyone a candidate for dental implants?	D1
3.	Is dental implant surgery safe?	D1, D4
4.	Is dental implant surgery painful?	D1, D4, D6
5.	Can I get dental implants during pregnancy?	D1
6.	Will the dental implant look and feel like my natural tooth?	D1, D4, D6
7.	Two of my teeth have become loose and fallen out. The doctor said it's due to periodontitis. Can I get dental implants?	D4
8.	How long after tooth extraction can I get dental implants?	D4
9.	One of my lower jaw molars was extracted, and the doctor suggested installing a dental implant. How long is the entire treatment cycle?	D4
10.	What are the common dental implant brands? How should I choose?	D1
11.	The doctor mentioned that I don't have enough bone for an implant and need a bone grafting surgery. What is this exactly?	D1, D4
12.	Are there any risks associated with bone grafting surgery?	D4, D6
13.	Can dental implants become inflamed?	D1, D5
14.	Is it safe to undergo CT scans and MRI after getting dental implants?	D1, D5
15.	Is it okay to undergo dental cleaning after getting dental implants?	D5
16.	Do I need to have regular follow-up after getting dental implants?	D5
17.	How long can I expect my dental implants to last?	D1, D5, D6
18.	How should I maintain and care after getting dental implant restorations?	D5
19.	Can I eat normally after getting an implant?	D5
20.	My dental implant has been in place for 1 year. During the review, the doctor said that '2mm of the alveolar bone around the implant has been absorbed.' What does this mean? What should I be aware of?	D1, D5

*Domains: D1, Education; D2, Prevention; D3, Diagnosis; D4, Treatment; D5, Management; D6, Support.
