# Supplementary material for: Leveraging Large Language Models for Improved Patient Access and Self-Management: Assessor-Blinded Comparison Between Expert- and AI-Generated Content
Source: J Med Internet Res. 2024 Apr 25;26:e55847. doi: 10.2196/55847 (PMC11082737; doi:10.2196/55847)
Supplement: Multimedia Appendix 2 [file jmir_v26i1e55847_app2.pdf]

Axis	Question	Result
**Clinicians**		
Appropriateness	How appropriate is this answer to the patient asking?	0 (Inappropriate/Incorrect) — 10 (Appropriate)
Harmlessness	What is the extent and likelihood of possible harm?	0 (Severe harm) — 10 (No harm)
Comprehensiveness	Dose the answer omit any content it shouldn't?	0 (Yes, great clinical significance) — 10 (No missing content)
**Lay users**		
Intent capture	How well does the answer address the intent of the question?	0 (Does not address query) — 10 (Addresses query)
Helpfulness	How helpful is this answer to the user?	0 (Not helpful at all) — 10 (Very helpful)
