# Supplementary figures and images for: Leveraging Large Language Models for Improved Patient Access and Self-Management: Assessor-Blinded Comparison Between Expert- and AI-Generated Content
Source: J Med Internet Res. 2024 Apr 25;26:e55847. doi: 10.2196/55847 (PMC11082737; doi:10.2196/55847)

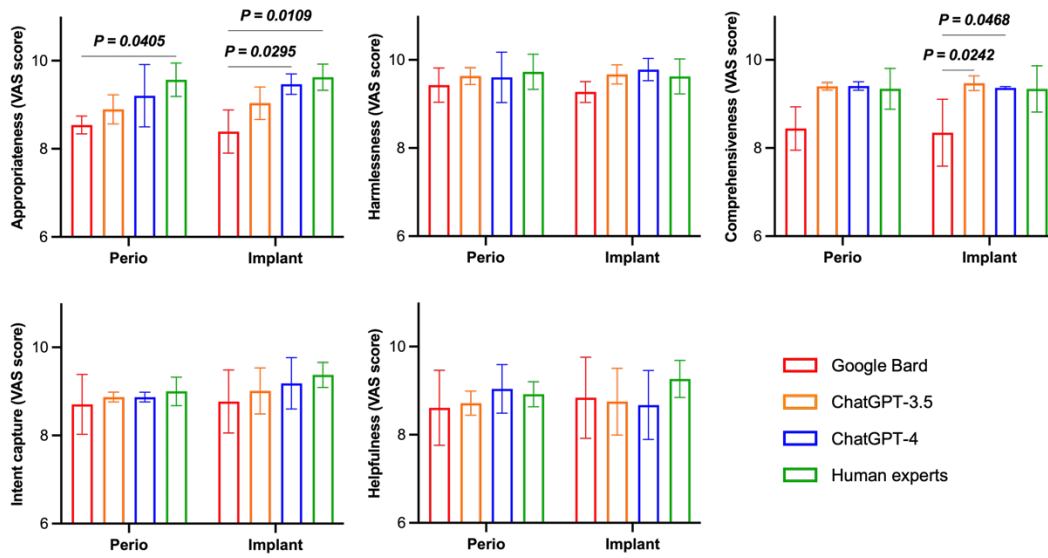

Supplement: Multimedia Appendix 3 [file jmir_v26i1e55847_app3.pdf]

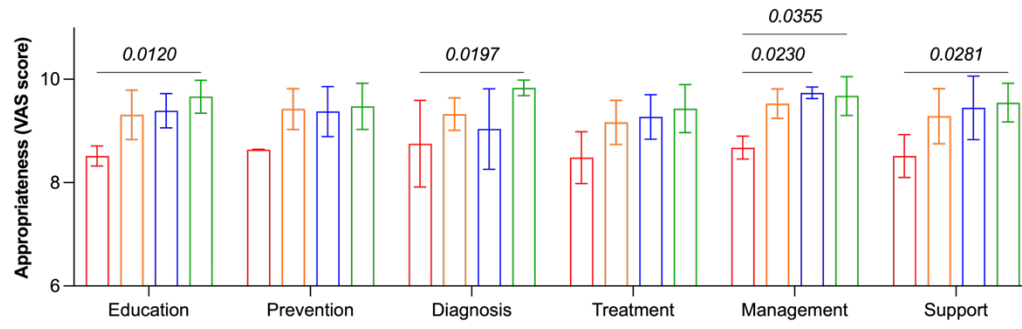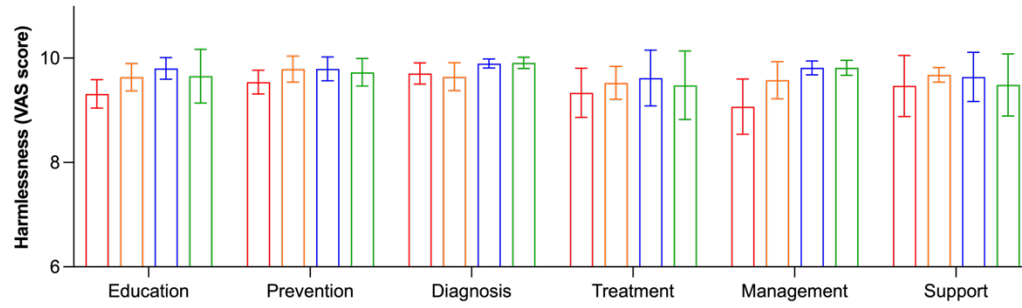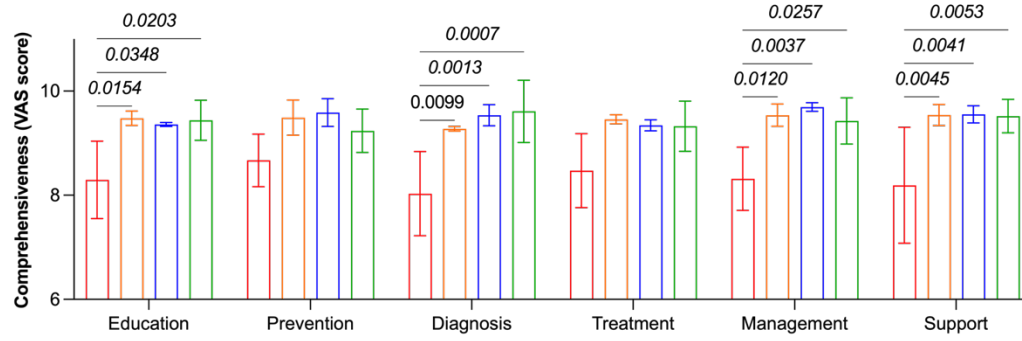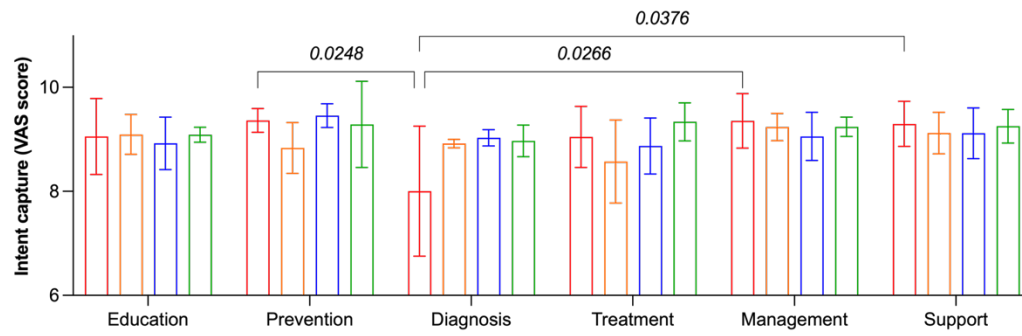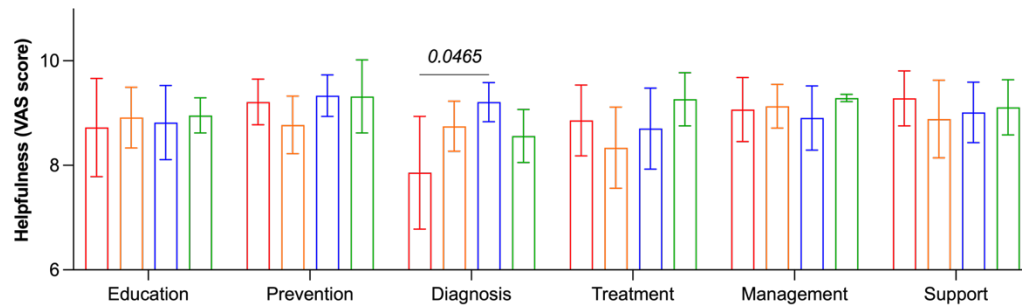

Supplement: Multimedia Appendix 4 [file jmir_v26i1e55847_app4.pdf]
